# Small Effects of Selective Migration and Selective Survival in Retrospective Studies of Fertility: Faibles effets de sélection de la migration et de la mortalité sur la fécondité dans les études rétrospectives

**DOI:** 10.1007/s10680-013-9293-6

**Published:** 2013-06-04

**Authors:** Gunnar Andersson, Boris Sobolev

**Affiliations:** 1 Department of Sociology, Stockholm University Demography Unit, SE-106 91 Stockholm, Sweden; 2 School of Population and Public Health, University of British Columbia, Vancouver, Canada

**Keywords:** Fertility, Selectivity, Retrospective data, Register data, Sweden, Fécondité, Sélection, Données rétrospectives, Données de registre, Suède

## Abstract

In this study, we assess the accuracy of fertility estimates that stem from the retrospective information that can be derived from an existing cross-sectional population. Swedish population registers contain information on the childbearing of all people ever registered as living in Sweden, and thus allow us to avoid problems of selectivity by the virtue of survival or nonemigration when estimating the fertility measures for previous calendar periods. We calculate two types of fertility rates for each year in 1961–1999: (i) rates that are based on the population that was living in Sweden at the end of 1999, and (ii) rates that also include information on people who had died or emigrated before the turn of the twentieth century. We find that the omission of information on individuals who had emigrated or died, as the situation would be in any demographic survey, most often have negligible effects on fertility measures. However, first-birth rates of immigrants gradually become more biased as we move back in time from 1999 so that they increasingly tend to over-estimate the true fertility of that population.

## Résumé

Dans cet article, nous évaluons l’exactitude des estimations de la fécondité à partir des informations rétrospectives issues de données transversales sur la population. Les registres de population suédois relèvent des informations sur toutes les personnes enregistrées en âge de procréer qui vivent en Suède, nous permettant ainsi d’éviter les problèmes de sélection induits par la survie ou la non-migration quand la fécondité est estimée pour des années antérieures. Nous calculons deux types de taux de fécondité pour chacune des années de la période 1961–1999: (i) des taux qui se rapportent à la population vivant en Suède à la fin de l’année 1999, et (ii) des taux qui incluent également des informations sur les personnes qui sont décédées ou ont émigré avant la fin du 20^ème^ siècle. Les résultats montrent que l’omission de l’information relative aux individus qui ont émigré ou sont décédés, ce qui serait le cas dans une enquête démographique, a des effets le plus souvent négligeables sur les mesures de fécondité. Cependant, en ce qui concerne les immigrantes, plus on s’éloigne dans le temps de l’année 1999, plus les taux de fécondité pour les naissances de rang 1 surestiment la fécondité réelle de cette population.

## Introduction

The purpose of this study is to provide an examination of the magnitude of the bias that may appear in fertility estimates that are based on retrospective information on childbearing gathered at a fixed point in time. Many studies of human fertility are based on survey data that are collected by asking respondents about their preceding histories of childbearing and about other related behaviors. Generally, such information is considered reliable since the birth of a child is such an important event in people’s lives that respondents will report it accurately. Normally, researchers raise doubts only about the accuracy of men’s reports on childbearing since they sometimes are found to underreport the existence of children who are fathered outside a stable union (Rendall et al. 1999; Greene and Biddlecom 2000). Further, the data collected in developing countries may be distorted by means of age heaping in demographic indicators. However, even if we restrict ourselves to the very reliable histories of childbearing as reported by women in developed countries, we may be faced with some problems if we try to estimate the measures of fertility for the population of a given area for periods preceding the survey date.

A bias in estimates may arise if the cross-sectional population of a specific area has had a different fertility behavior than people who previously lived there but had left it at the time of data collection. Literature on the fertility of migrants, for example, suggests that they often display a pattern of relatively low fertility before migration, but of elevated fertility shortly after migration (Goldstein and Goldstein 1981; Ford 1990; Alders 2000; Andersson 2004; Toulemon and Mazuy 2004; Andersson and Scott 2005; Kulu 2006; Milewski 2007; Parrado 2011). Such a pattern arises if the childless are more prone to migrate than parents are, and if family formation and childbearing typically occur after a (long-distance) migration. Since previous out-migrants from an area do not show up in a survey that is based on the cross-sectional population of that area, their potential low-fertility behavior will be absent in the survey data while instead the high-fertility behavior of newly arrived in-migrants in the area is covered properly. If there are similar selection effects in reproductive histories by the virtue of the survival of women, we will also be faced with a bias arising from the omission of individuals who had died before the data were collected. Doblhammer (2000) shows that childless women have a slightly higher mortality at ages above 50 than mothers have, which suggests that such selection effects indeed might appear. Again, the omission of data on deceased individuals from any sample would then result in an over-estimation of the previous fertility level in the area since the persons who have been left out are suspected to have had a somewhat lower fertility than the surviving population. Nevertheless, any effects of that kind must be very small since the relationship between reproductive behavior and mortality is quite weak (for a review, see Hurt et al. 2006; see also Jaffe et al. 2009).

Normally, it is difficult to assess the sign of any selection effects of the kind we discussed above. In the present study, however, we are indeed able to provide evidence of the existence and magnitude of such effects by using a data set that contains information on the childbearing histories of an existing cross-sectional population and, in addition, the corresponding information on people who previously had lived in the area under investigation but had died or emigrated. For this purpose, we use population-register data of Sweden, which cover the childbearing, mortality, and migration histories of all women who had ever lived in that country from 1961 to 1999. Since data on persons, who no longer live in Sweden, are saved in the register records, we are able to perform a calculation of fertility measures over the period 1961–1999, as they would have appeared in a prospective study on fertility starting in 1961. As an experiment, we also choose to exclude all the information that refer to people who no longer lived in Sweden at the end of 1999, as the situation would have been if we had conducted a retrospective survey at that time. By comparing the fertility estimates that are based on (i) the prospective study design and (ii) the retrospective design, we are able to examine if the latter type of study produces fertility measures that are different from those stemming from the complete information of the prospective study. If any bias appears, we expect it to become more important as we move back in time from our simulated survey date of the last day of December, 1999, and we report the relative magnitude of any such bias. We provide separate analyses of the bias that may appear when the Swedish-born population is studied and when the fertility of foreign-born immigrants is considered.

## Data and Methods

Our data stem from the Swedish population-registration system, which provides very reliable information on the demographic histories of Swedish people with the help of a unique identifying code for each individual ever registered as living in Sweden and due to an efficient coverage of vital events occurring in that country. Our extract of data contains information on childbearing, mortality, and migration of all women born in Sweden from 1925 onwards (who were either registered in the census of 1960 or born after that census) as well as the corresponding information on women born abroad from 1925 onwards who had ever lived in Sweden between 1961 and 1999.^1^ Residence in Sweden amounts to de jure residence. The registration of immigration or emigration requires that the migrant intends to stay in Sweden or abroad for at least 1 year. Temporary migrants to Sweden and people without residence permit are not covered by Swedish population registers, nor are any births to such persons. The data cover resident women’s full childbearing histories until a death, an emigration, or the 31st of December, 1999—whichever comes first.

Our study population is presented in Table 1, which gives the total number of women by three very broad country-groups of origin. The vast majority of women are of course born in Sweden but the data also contain large numbers of immigrants—of whom about 40 % stem from the neighboring Nordic countries. The two middle columns of Table 1 report the number of women in the population who emigrated from Sweden or died in Sweden in 1961–1999, which means that they no longer lived there at the end of 1999. The immigrant population is relatively young, so the exclusion of immigrants by the cause of mortality is fairly unimportant. Instead, we note that large numbers of immigrants have again emigrated from Sweden. Almost a third of immigrants from the neighboring Nordic countries, and more than a fifth of immigrants from non-Nordic countries had left Sweden by the end of 1999. This is not particularly remarkable since the return migration is a typical feature of all types of migratory streams. Nevertheless, it points to the need for having access to longitudinal information on immigrants as well as emigrants, if one wants to have a full picture of the demographic behavior of any mobile population segment in a country.

**Table 1:** Number of women in our data who ever lived in Sweden between 1961 and 1999, by country group of birth, and the number of these women who no longer lived in Sweden by the end of 1999 because of out-migration or death

Country of birth	Study population	Died before year 2000	Emigrated before 2000	Population in Dec. 1999	Percent retained (%)
Sweden	2,973,000	117,000	69,000	2,787,000	94
Other Nordic	197,000	8,000	59,000	129,000	66
Non-Nordic	313,000	6,000	64,000	242,000	77

With our data, we calculate relative risks of childbearing by calendar year from 1961 to 1999 for women at different parities. In our event-history analyses, we control for the effect of the age of a woman and the age of any youngest child of hers. We estimate separate models of first-birth risks for women of ages 16–26, and for women of ages 30–45, since we know that the trends in childbearing have been quite different for childless women in the younger and the older age brackets (Andersson 1999). We present separate sets of parity-specific fertility measures for women born in Sweden and for women born abroad.

We calculate the fertility rates in two rounds. First, we use the full information of all women available in the data for our calculations. Second, we exclude women who had died or emigrated from Sweden before the turn of the century. This gives us a data set with information on the childbearing of the cross-sectional population on December 31, 1999—just like we would get if we had conducted a survey at that time. We use this latter data with retrospective-type information only, to calculate the same sets of fertility rates by calendar year as we produced in our first round of calculations. The purpose of this procedure is to relate the fertility measures of the second round to those of the first in order to see whether we can find any systematic deviation in apparent risk patterns. We report the relative deviation in fertility rates at various time horizons back in time from our simulated interview date in order to see how far back one typically can rely on retrospectively reported data without facing any serious problems of bias in fertility estimates of different groups of women. We use the Genmod module of SAS in order to calculate our fertility measures. For a further description of our data and the type of models we have estimated, see Andersson (1999).

## Results

**Fig. 1:**
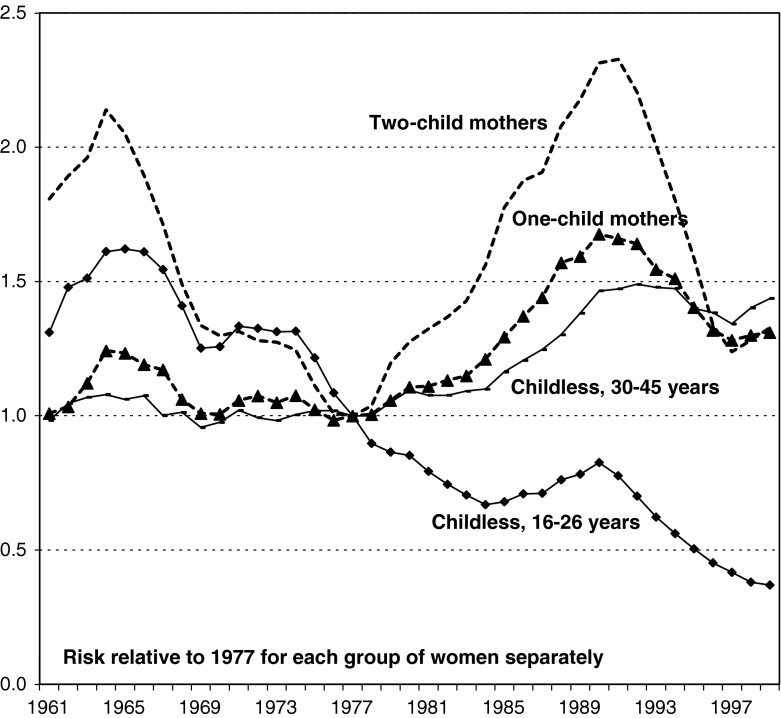
Relative risk of childbearing by calendar year, Swedish-born women in Sweden 1961–1999, standardized for age of woman and any youngest child

As an introduction, we present the relative risks of childbearing by calendar year for childless younger women, childless women at ages 30–45, one-child mothers, and two-child mothers who were all born in Sweden, with a separate curve for each category of women (Fig. 1). These risks are based on the full information on childbearing available in the register data. Our fertility measures are given on a relative scale for each group of women separately, so we get a good picture of changes over time in the propensity to give birth, but no information on differences in fertility levels between the different categories of women. Evidently, fertility in Sweden has fluctuated considerably during our study period, with important turning points occurring in 1964, 1977, 1984, 1990, and 1997. We do not intend to discuss the background of these developments in this presentation, but refer instead to Hoem and Hoem (1996) and Andersson (1999) for a more detailed discussion of patterns in childbearing in Sweden during our study period; for more recent updates on fertility trends, see Andersson and Kolk (2011). Trends in childbearing in Sweden of foreign-born women very much resemble those of the Swedish-born, even though the fertility levels of the foreign-born in many cases are higher than those of the Swedish-born (not shown here). For a description and further analyses of patterns in childbearing of the immigrant population in Sweden, see Andersson (2004) and Andersson and Scott (2005, 2007).

In Table 2, we present the main results of our investigation with a comparison of childbearing risks of Swedish-born women as calculated from our two data sets. We report the relative deviation in estimated risks for the “retrospective” study as compared to those of the “prospective” study for different calendar years prior to 1999, i.e., at different time periods from our simulated data collection. Separate columns give the results for the various parity and age groups we examine.

**Table 2:** Relative bias in retrospectively collected fertility data by period before data collection, parity, and age group of women (in percent)

Years prior to study	First births, 16–26 years	First births, 30–45 years	Second births	Third births
1 (1998)	0	0	0	0
2 (1997)	1	0	0	0
3 (1996)	1	1	0	0
5 (1994)	2	1	0	0
7 (1992)	2	1	0	0
10 (1989)	2	1	1	0
15 (1984)	2	2	1	0
20 (1979)	2	3	1	0
25 (1974)	2	5	1	0
30 (1969)	3	6	2	1
35 (1964)	3	7	2	0

Comparison of childbearing rates of women born in Sweden: rates from retrospectively collected data related to rates from the full data set

Evidently, a retrospective gathering of data results in a minor overestimation of fertility measures as we move back in time from the year when the data were collected (1999). However, in most cases these effects are of no importance. For younger childless women, we only get a bias of around two percent when we move some 5 years back in time and we do not get a bias higher than three percent even if we move several decades back in time. When we estimate fertility measures for mothers, we find that the bias from any selection due to survival or emigration is virtually non-existent. The only case where a bias really appears is when we estimate fertility rates for childless women at ages above 30, but this bias only turns out to be visible if we move some 20–25 years back in time. In order to check whether the bias in first-birth rates of older childless women arises from selective mortality or from selective migration, we re-estimate our “retrospective” models leaving out only one group of absent (deceased or emigrant) individuals at a time while keeping the information on the others in our data (not shown here). Such an exercise reveals that the bias in fertility estimates of older women stems almost entirely from differential mortality by the motherhood status of the elderly.

In conclusion, the general picture from our experiment is that the effects of selectivity by virtue of survival or of emigration is quite unimportant when we estimate fertility measures from retrospectively collected data for a local population. The only bias we found appeared when we estimated fertility measures for women who were childless in their 30s or 40s some 20 years back in time. In this case, differential mortality by changes in motherhood status at the older ages caused a bias in fertility estimates. However, this category of women is seldom the target of conventional fertility studies so our finding should not cause too much of a worry for researchers who work with retrospective data.

In Table 3, we proceed by presenting the results of the corresponding examination of data for foreign-born women in Sweden, with results given for fertility estimates of immigrant women from the non-Nordic countries. As this is a much more mobile group of people than the native population, we might expect greater effects of a selection from the remaining cross-sectional population of December 31, 1999—and this is indeed what we find. Retrospective first-birth rates of foreign-born women increasingly tend to overestimate the childbearing of the immigrant population in Sweden as we move back in time from 1999. The effects are already visible a few years prior to the date of data collection, and our fertility measures overestimate the true first-birth fertility by some ten percent at 15–20 years prior to the simulated survey date. By contrast, if we only study the childbearing behavior of immigrant *mothers*, we find that the retrospectively collected data cover the childbearing dynamics very well. We assume that the bias we find for the childless women is mainly due to differential emigration by motherhood status and we confirm this hypothesis by estimating models where we leave out only the emigrated women from our data while retaining the deceased ones (not shown).

**Table 3:** Relative bias in retrospectively collected fertility data by period before data collection, parity, and age group of women (in percent)

Years prior to study	First births, 16–26	First births, 30–45	Second births	Third births
1 (1998)	1	1	0	1
2 (1997)	2	2	1	0
3 (1996)	3	4	1	1
5 (1994)	4	9	1	1
7 (1992)	6	8	2	2
10 (1989)	7	4	2	2
15 (1984)	9	8	1	0
20 (1979)	10	10	0	4
25 (1974)	11	10	−1	1

Comparison of childbearing rates of foreign-born women from non-Nordic countries: rates from retrospectively collected data related to rates from full data

To summarize, the results of the second part of our experiment were a bit more discouraging than those of our examination of fertility estimates for the native population were. Evidently, the propensity of childless immigrants to re-emigrate is higher than the corresponding propensity of immigrant mothers and this selectivity in migration behavior causes a bias in any first-birth estimates that are based solely on the remaining immigrant population of an area. The omission of substantial numbers of childless emigrants from our data results in an overestimation of the fertility of the immigrant population in Sweden. However, if we avoid stretching our fertility analysis too far back in time, we can also avoid any unacceptable overestimation of the fertility of the immigrant population.

## Conclusions

With our analysis, we have managed to get a clear picture of the reliability of fertility measures that are based on retrospectively collected data when it comes to their ability to describe the childbearing of a population of a given geographical area in calendar periods prior to data collection. We used population-register data of Sweden in order to simulate a collection of data at a given point in time. We compared the fertility estimates from such a retrospective data collection to fertility rates that also pick up the childbearing behavior of people who had left the area under investigation before the time of data collection. Our results are rather encouraging because they demonstrate that the omission of individuals who emigrated or died rarely results in more than a minor overestimation of fertility rates in periods before the data collection. However, the reliability of retrospectively collected data mainly holds when we describe the behavior of a population with moderate or low levels of out-migration. If we focus on the more mobile immigrant population, we actually face some problems of selectivity in the data that only contain information on the immigrants who remained in the country of immigration. Most immigrant populations display relatively high levels of return migration, so any demographic estimate of such a population easily risks being affected by various types of selective out-migration.^2^ To minimize such problems, we recommend that retrospectively collected data on the childbearing behavior of immigrants should mainly be analyzed for relatively short time periods before the data collection.

The present study was based on data for Sweden, but the general patterns likely hold also for other countries across Europe and beyond. In relative terms, Sweden has somewhat high levels of immigration and fertility and fairly low levels of mortality. Recent research from Europe and North America shows that patterns in interdependencies between migration and childbearing appear quite similar across contexts (see references in Sect. 1). For example, family formation and migration tend to be interrelated processes and migration rates are typically lower for parents than for nonparents. For relatively mobile populations, such patterns and differentials in behavior easily translate into selectivity issues when we estimate fertility measures for groups of remaining non-(out) migrants. For less mobile populations, these issues are of less concern.

Our contribution had a methodological focus: it examined the bias that may appear when using cross-sectional and retrospective data to estimate fertility rates for populations that are exposed as well to the forces of mortality and migration. Our main conclusion was reassuring to analysts involved in such estimations, but we also highlighted the challenges involved in analyzing longitudinal data for populations that distribute their life course spells and vital events across different geographies. Evidently, research on life course dynamics that involve migrants and migration need to take issues of both space and time into serious consideration. For future research it would be interesting to examine the extent to which the relationships we exposed differ by various socio-economic and socio-demographic characteristics of the sub-populations.

## Footnotes

1 The data on immigrants only cover women who migrated to Sweden at ages younger than 35. This age limit reduces the possible problem of omitting from the birth records children who never joined their immigrant mothers to live in Sweden. We do, however, keep the vast majority of immigrant women in our study population since most of them arrived at young ages. For further information on the data on immigrants, see Andersson (2004).

2 In addition, the possibility of under-reporting of emigration events may cause problems when producing register-based demographic indicators for a population with much undocumented emigration (for evaluations of Swedish register data, see Qvist, 1999). This problem does not interfere very much with the present evaluation: non-registered emigrants would appear in the denominators of both sets of fertility indicators used in our comparisons.
